# Supplementary material for: Mass Spectrometric Identification of Licania rigida Benth Leaf Extracts and Evaluation of Their Therapeutic Effects on Lipopolysaccharide-Induced Inflammatory Response
Source: Molecules. 2022 Sep 23;27(19):6291. doi: 10.3390/molecules27196291 (PMC9571113; doi:10.3390/molecules27196291)
Supplement: Supplementary file 1 [file molecules-27-06291-s001.zip › molecules-1901650-Supplementary Figure S1.pdf]

Supplementary material

Figure S1. Comparison between library GNPS and query spectra of phytocomponents identified in *Licania rigida Benth* by LC-MS/MS analyses.

Comparison between library GNPS (bottom) and query spectra phytocomponents identified in **AELR** (top). The structure of the phytocomponent identified is represented.

Gallic acid

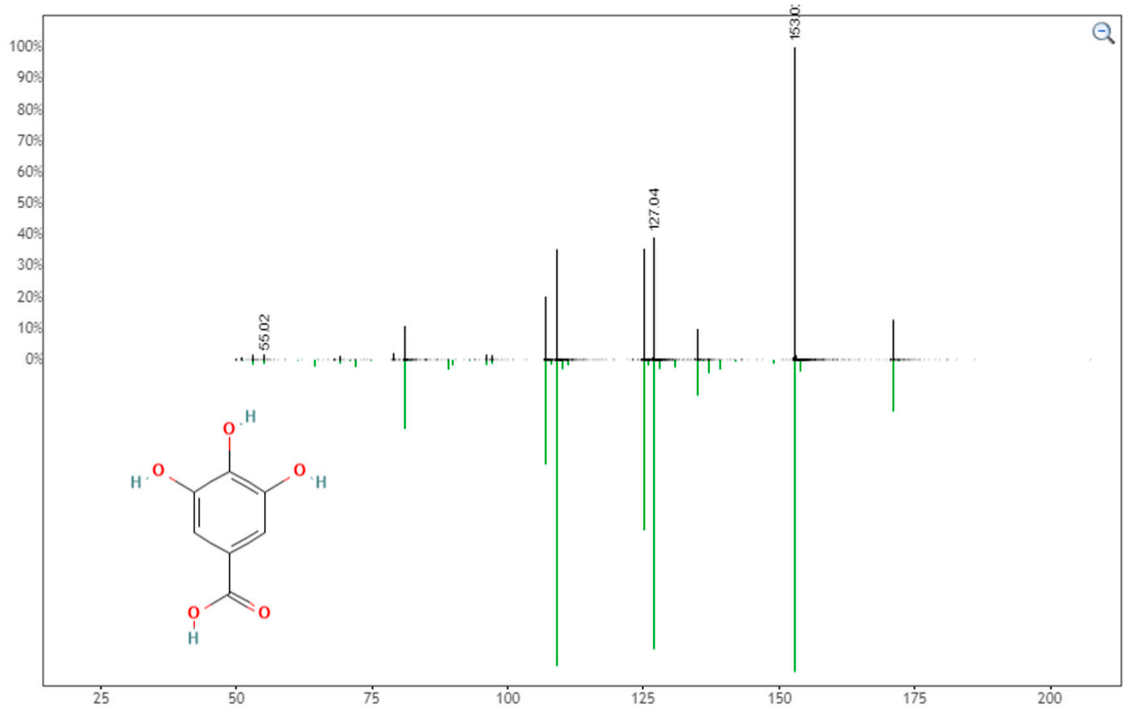

5_-Deoxy-5_-(methylsulfinyl)adenosine

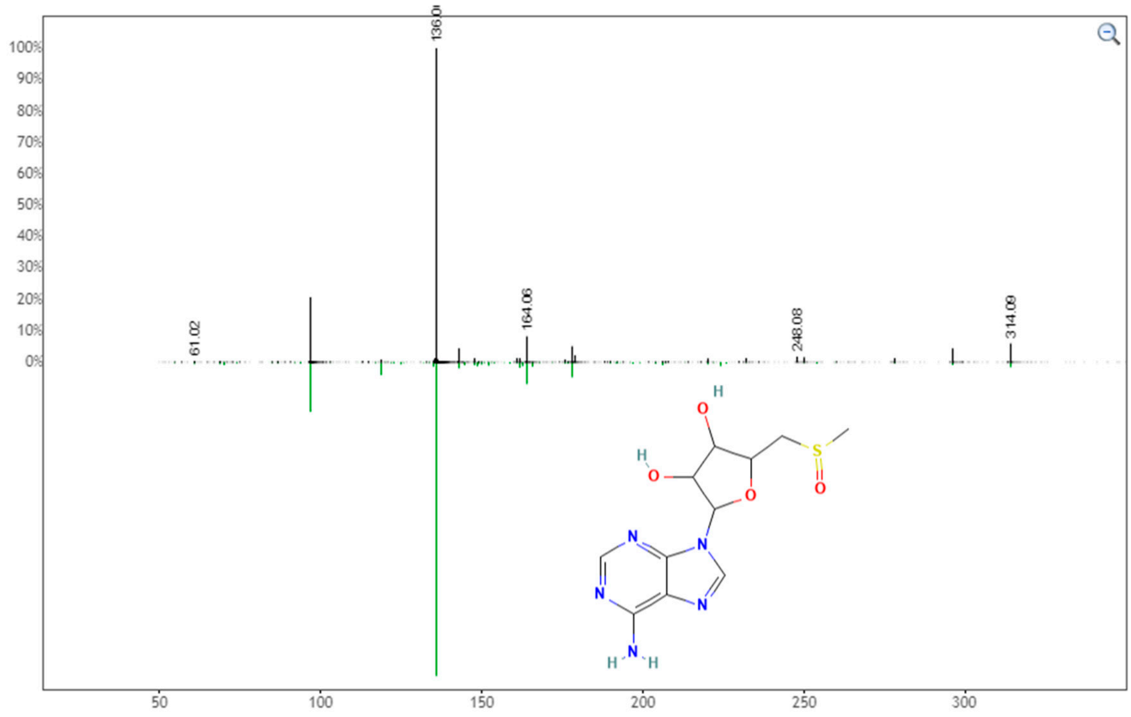

Adenosine Monophosphate

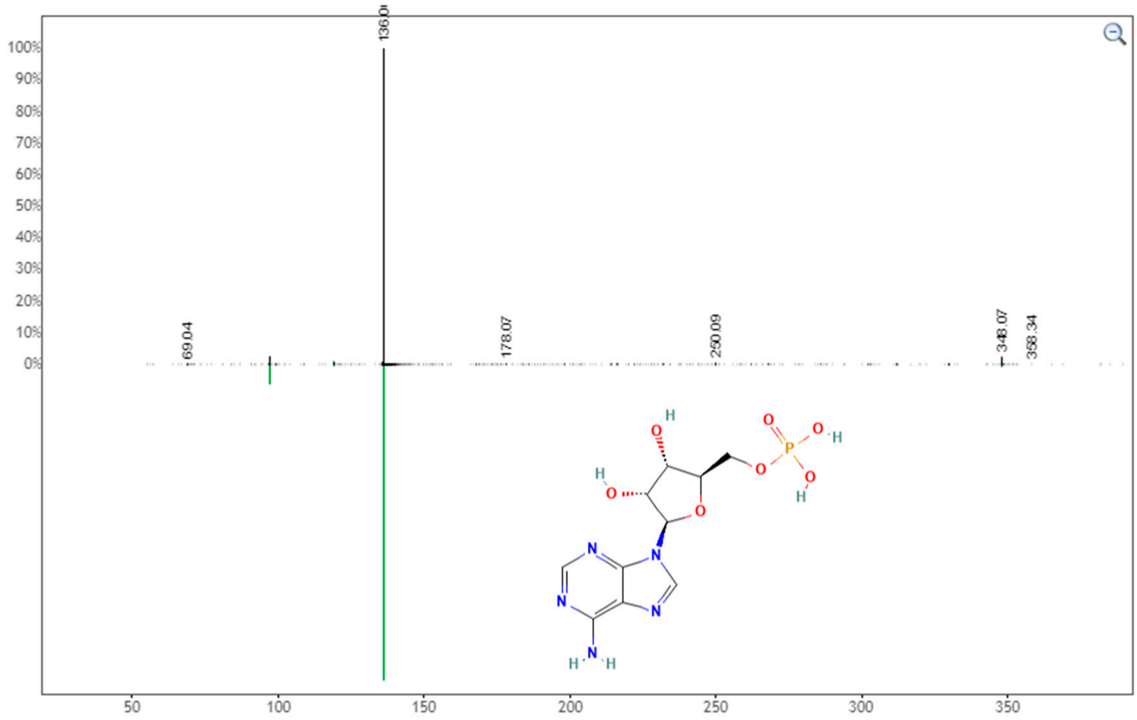

Ellagic acid

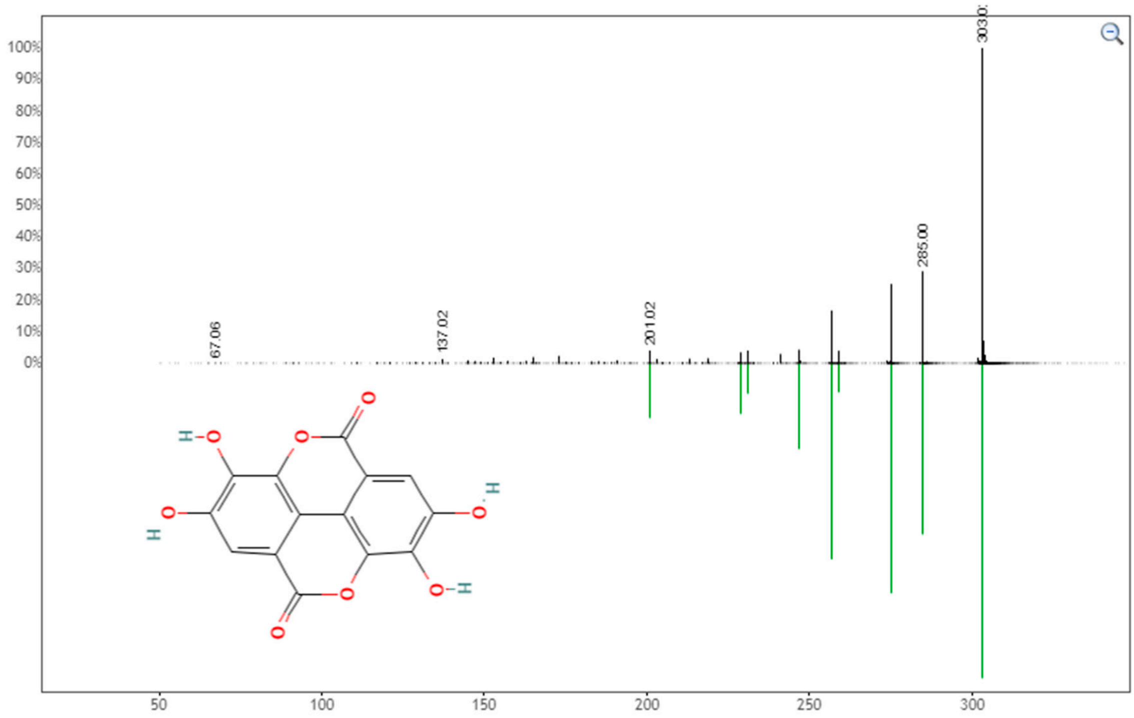

Comparison between library GNPS (bottom) and query spectra phytocomponents identified in **HELR** (top). The structure of the phytocomponent identified is represented.

DL-Indole-3-lactic acid

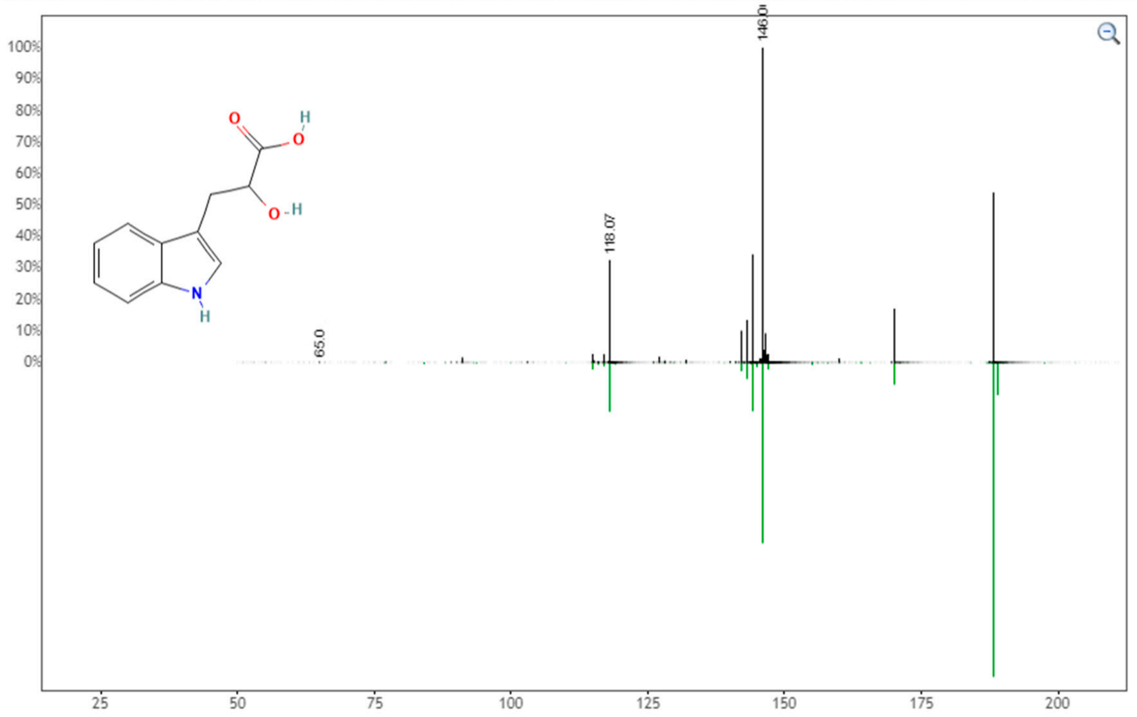

Isovitexin

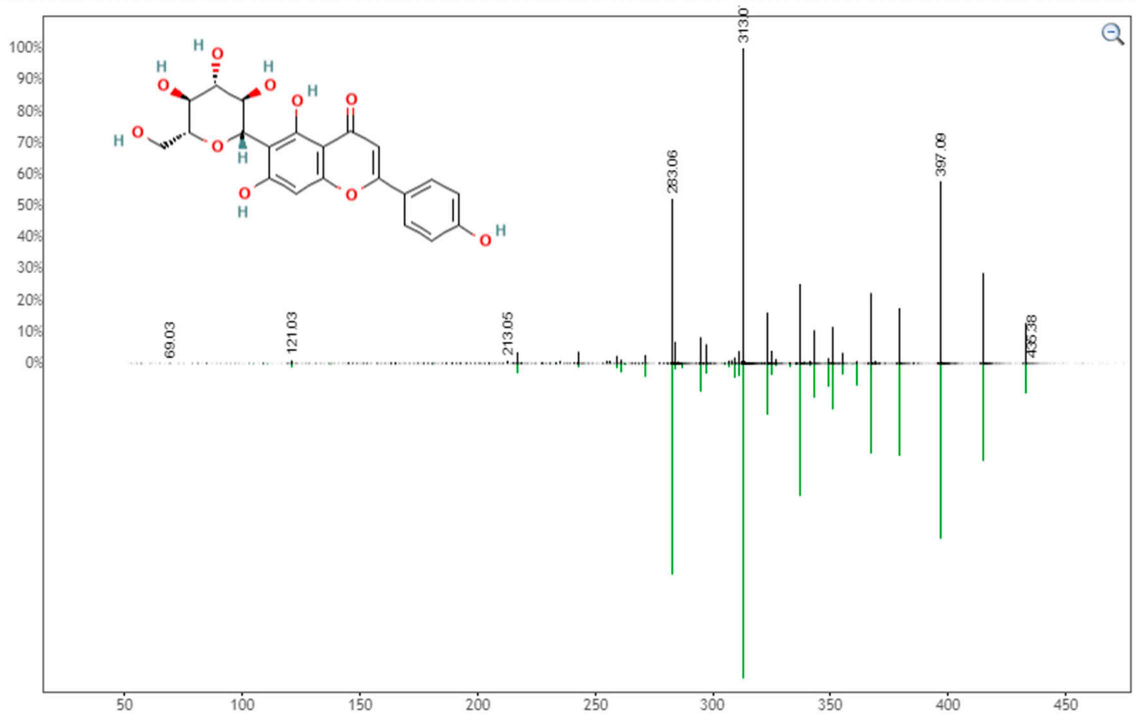
100%
90%
80%
70%
60%
50%
40%
30%
20%
10%
0%
313.0
283.06
397.09
69.03
121.03
213.05
435.38
50
100
150
200
250
300
350
400
450

2,6-Xylidine

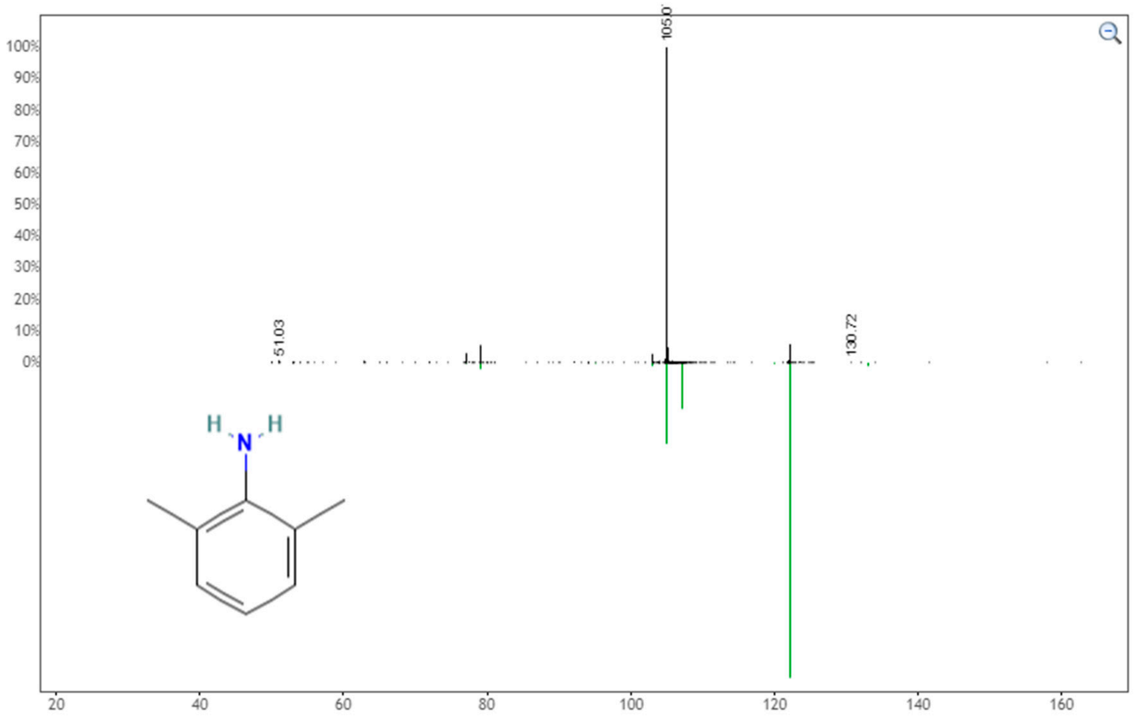
100%
90%
80%
70%
60%
50%
40%
30%
20%
10%
0%
105.0
51.03
130.72
20
40
60
80
100
120
140
160

Gallic acid

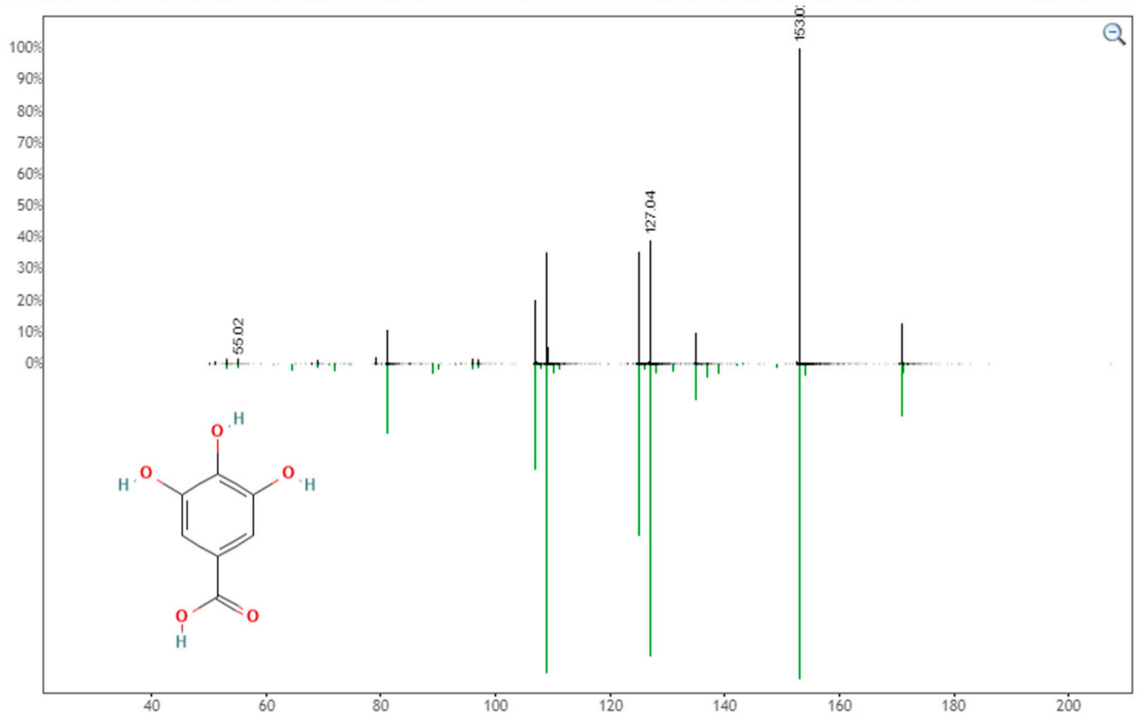

Ferulate

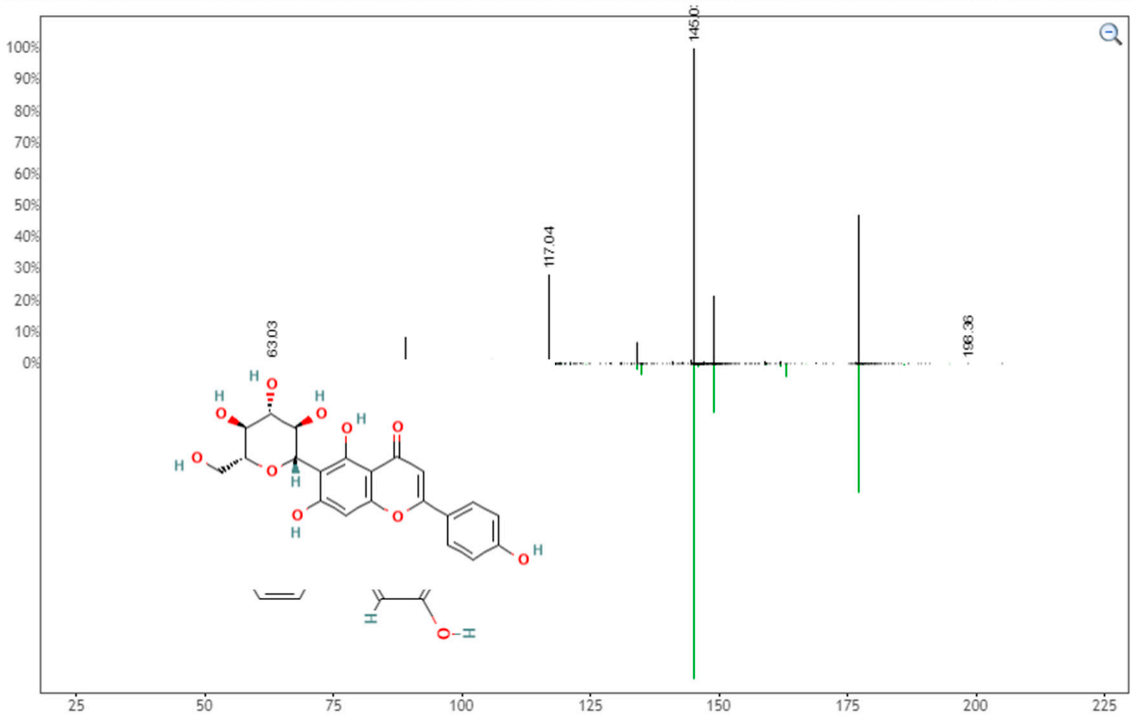

Phenylalanine

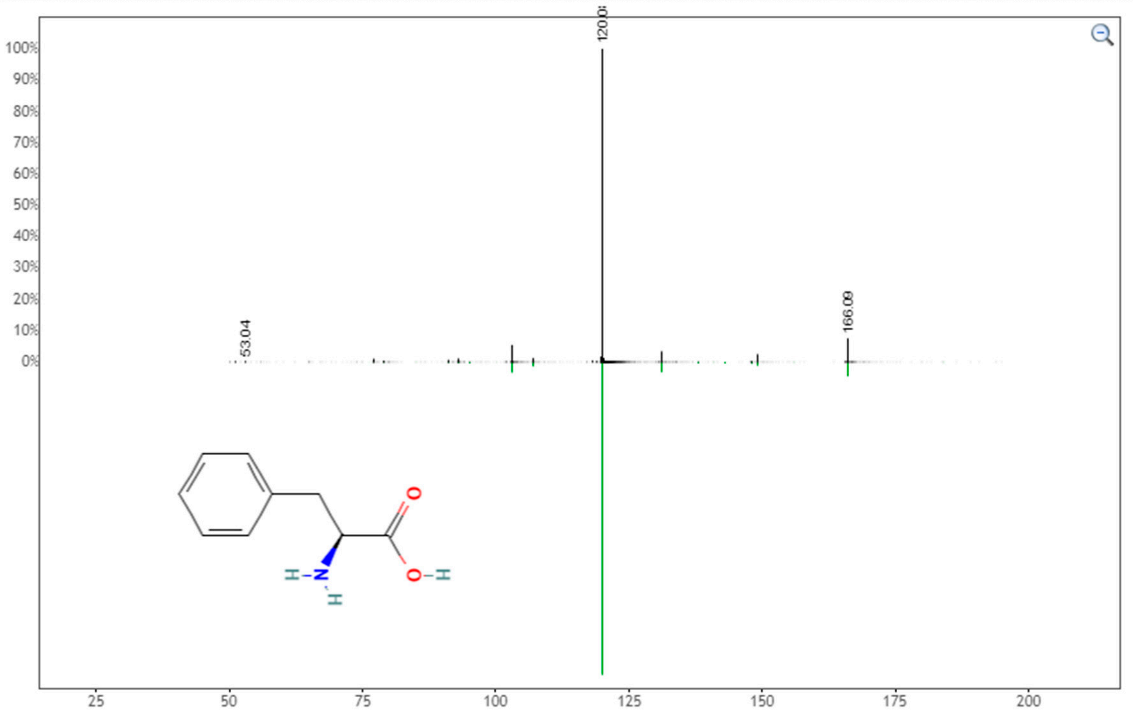

Pheophorbide A

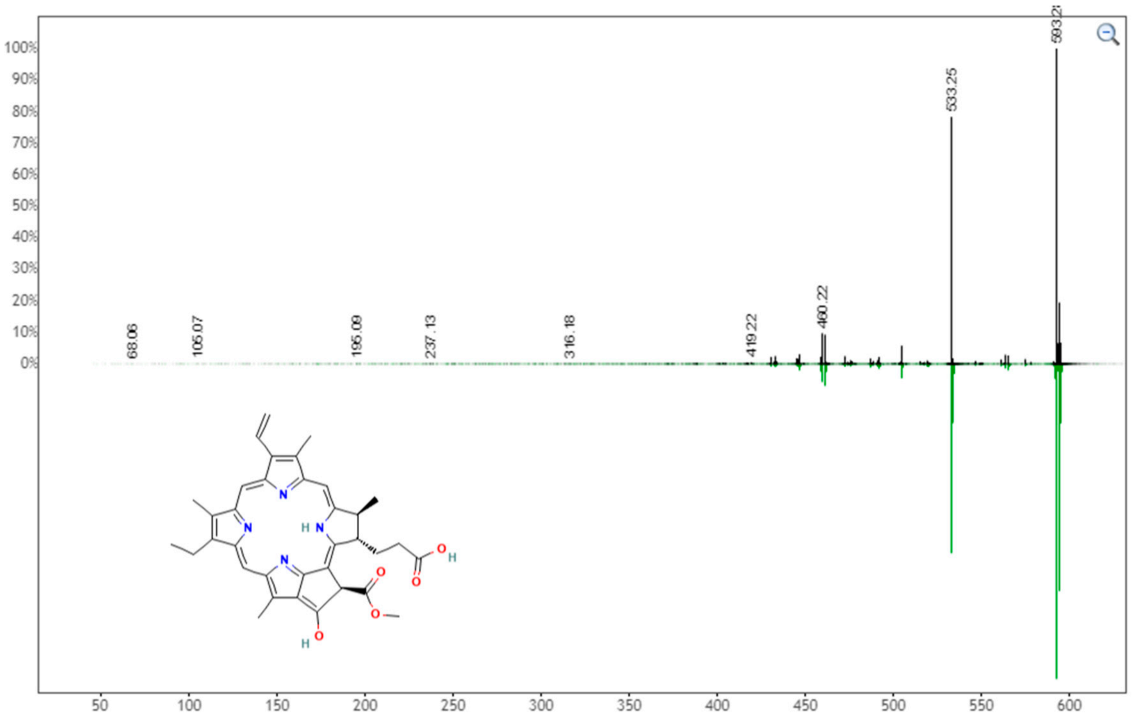

4-AMINOBUTANOATE

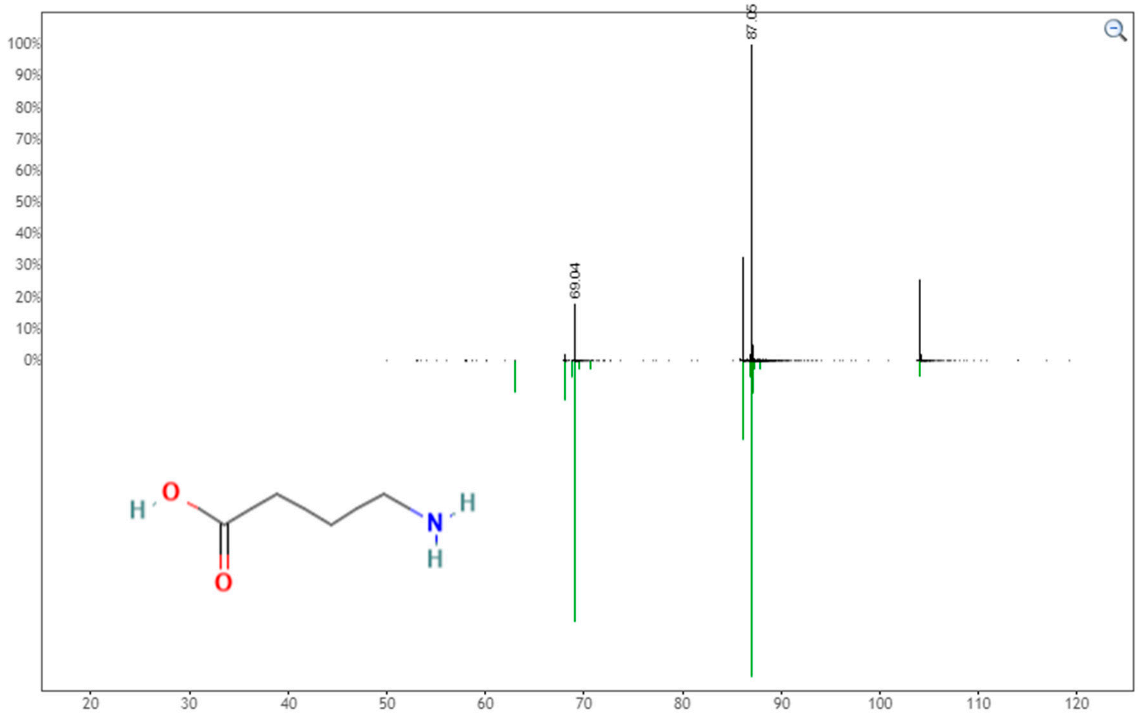
87.05
69.04
100%
90%
80%
70%
60%
50%
40%
30%
20%
10%
0%
20
30
40
50
60
70
80
90
100
110
120

Pyridoxine

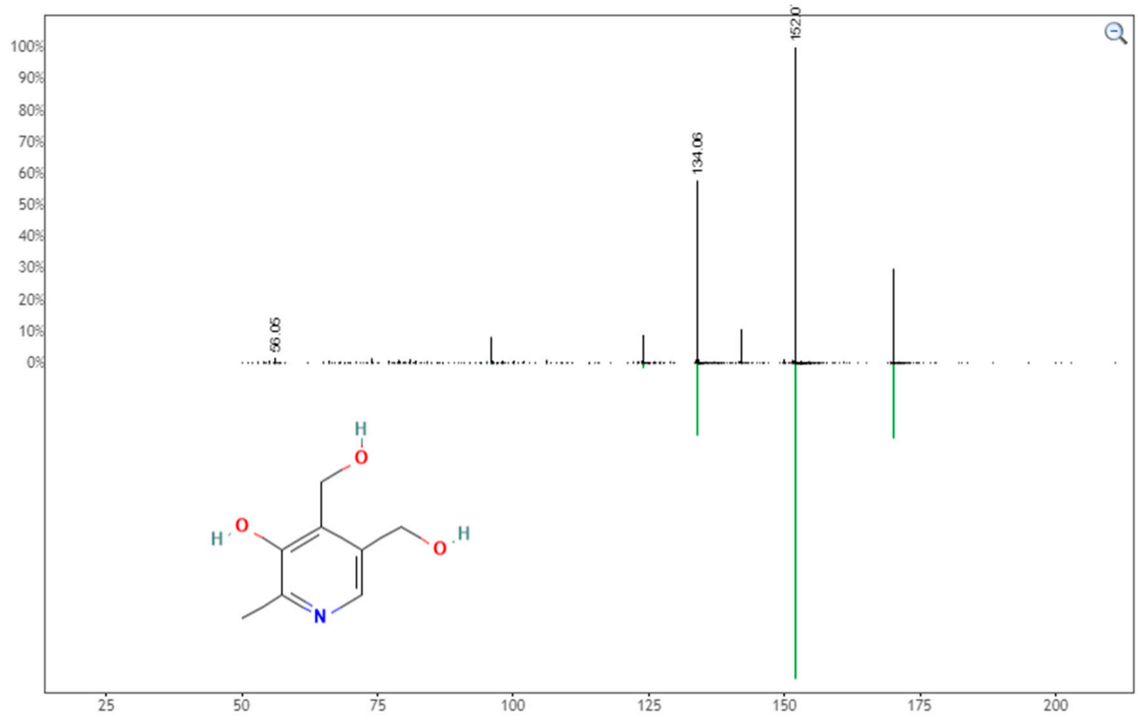
152.0
134.06
56.05
100%
90%
80%
70%
60%
50%
40%
30%
20%
10%
0%
25
50
75
100
125
150
175
200
